# Poisoning by Pseudo-Homœopathy

**Published:** 1874-05

**Authors:** 


Poisoning by Pseudo-Homœopathic Preparations.—At a recent meeting of the Clinical Society of London, Dr. George Johnson reported three cases of poisoning, from small doses of the "Concentrated Essence of Camphor," now extensively employed by the followers of Hahnemann. Dr. Johnson called attention to the notorious fact that, in England, the homœopaths have lately made a change of base, "passing from the irrational and ludicrous extreme of infinitesimal dilutions to the dangerous extreme of the greatest possible concentration of active and poisonous drugs." In the discussion which followed the reading of this paper, several other cases were cited of analogous malpractice on the part of these charlatans, who, being unwilling to publicly renounce the absurdity of infinitesimal doses, have recourse to these concentrated tinctures, in order to produce an appreciable effect by very minute doses. The British Medical Journal, in commenting upon this exposure, finds additional evidence that homœopathy, "which had begun as a delusion, is now rapidly ending as a fraud."

